# Effects of Thyroxine Exposure on Osteogenesis in Mouse Calvarial Pre-Osteoblasts

**DOI:** 10.1371/journal.pone.0069067

**Published:** 2013-07-23

**Authors:** James J. Cray, Kameron Khaksarfard, Seth M. Weinberg, Mohammed Elsalanty, Jack C. Yu

**Affiliations:** 1 Department of Oral Biology, Georgia Regents University, Augusta, Georgia, United States of America; 2 Department of Orthopaedic Surgery, Georgia Regents University, Augusta, Georgia, United States of America; 3 Department of Orthodontics, Georgia Regents University, Augusta, Georgia, United States of America; 4 Department of Surgery, Division of Plastic Surgery, Georgia Regents University, Augusta, Georgia, United States of America; 5 Institute for Regenerative and Reparative Medicine, Georgia Regents University, Augusta, Georgia, United States of America; 6 Departments of Oral Biology, Anthropology, and Orthodontics, University of Pittsburgh, Pittsburgh, Pennsylvania, United States of America; 7 Departments of Oral and Maxillofacial Surgery and Cellular Biology and Anatomy, Georgia Regents University, Augusta, Georgia, United States of America; Georgia Regents University, United States of America

## Abstract

The incidence of craniosynostosis is one in every 1,800–2500 births. The gene-environment model proposes that if a genetic predisposition is coupled with environmental exposures, the effects can be multiplicative resulting in severely abnormal phenotypes. At present, very little is known about the role of gene-environment interactions in modulating craniosynostosis phenotypes, but prior evidence suggests a role for endocrine factors. Here we provide a report of the effects of thyroid hormone exposure on murine calvaria cells. Murine derived calvaria cells were exposed to critical doses of pharmaceutical thyroxine and analyzed after 3 and 7 days of treatment. Endpoint assays were designed to determine the effects of the hormone exposure on markers of osteogenesis and included, proliferation assay, quantitative ALP activity assay, targeted qPCR for mRNA expression of *Runx2*, *Alp, Ocn*, and *Twist1*, genechip array for 28,853 targets, and targeted osteogenic microarray with qPCR confirmations. Exposure to thyroxine stimulated the cells to express ALP in a dose dependent manner. There were no patterns of difference observed for proliferation. Targeted RNA expression data confirmed expression increases for *Alp* and *Ocn* at 7 days in culture. The genechip array suggests substantive expression differences for 46 gene targets and the targeted osteogenesis microarray indicated 23 targets with substantive differences. 11 gene targets were chosen for qPCR confirmation because of their known association with bone or craniosynostosis (*Col2a1, Dmp1, Fgf1, 2, Igf1, Mmp9, Phex, Tnf, Htra1, Por,* and *Dcn*). We confirmed substantive increases in mRNA for *Phex*, *FGF1, 2, Tnf, Dmp1, Htra1, Por, Igf1* and *Mmp9*, and substantive decreases for *Dcn*. It appears thyroid hormone may exert its effects through increasing osteogenesis. Targets isolated suggest a possible interaction for those gene products associated with calvarial suture growth and homeostasis as well as craniosynostosis.

## Introduction

The thyroid hormones, triiodothyronine (T3) and thyroxine (T4) are essential for the growth and maintenance of all cells and organ systems in the body. Recent research has implicated maternal thyrotoxicosis, hyperthyroidism that results from increased thyroid hormone synthesis and secretion during pregnancy, as a risk factor for craniofacial birth defects. Thyrotoxicosis is estimated to occur in 1 in every 500 pregnancies and results from maternal hyperthyroidism accompanying thyroid hormone replacement for hypothyroidism [1], maternal Graves Disease [1], [2], [3], [4] or the presence of thyrotoxic goiter [1], [4], [5].

The Center for Disease Control National Birth Defects Prevention Study (CDC NBDPS) has identified maternal thyroid disease as a risk factor for craniosynostosis with an adjusted odds ratio of 2.47 [3]. Craniosynostosis is a pathological condition in which premature fusion of one or more of the calvarial sutures occurs before the cessation of brain growth. Craniosynostosis itself has an estimated birth prevalence of 1 case per 1,800–2,500 live births and involves the overgrowth of bone (synostosis) at the osteogenic fronts of the developing cranial bones [6]. The suture, a region of undifferentiated non-bony tissue, normally allows for prenatal and postnatal brain growth. When craniosynostosis is part of a syndrome, it is associated with facial, limb, ear, or heart malformations [7], [8], but the disorder most often appears in isolation (nonsyndromic) [9]. There is significant morbidity due to craniosynostosis resulting in various secondary effects including secondary fusion of other sutures, altered cranial base growth, and midface growth discrepancies [10], elevated intracranial pressure [6], altered intracranial volume [6], [11], and dilation of the subarachnoid spaces [12]. Such events may result in optic nerve compression, papilledema, and if left uncorrected, may lead to optic atrophy, blindness [13], cognitive disabilities, and mental retardation [14], which pose extensive, costly, and recurrent clinical and surgical management problems [15], [16].

During fetal development, thyrotropin-releasing hormone (TRH) and thyroid-stimulating hormone (TSH) are secreted beginning at 18–20 weeks gestation. These hormones are produced in the anterior pituitary gland and cause the follicular cells to take up iodinated thyroglobulin by endocytosis from the lumen of each thyroid follicle and then submit to lysosomal digestion. Two products result from the proteolysis, T3 and T4. T4 is more abundant making up about 90% of circulating hormone throughout life. Most T4 is converted to T3, a more rapid and potent hormone which remains at low levels until 30 weeks gestation. The fetus is self-sufficient concerning thyroid hormone, creating a relatively strong physiological protection against maternal hypothyroidism, however the hormone does cross the placental barrier [17], [18]. During postnatal growth, circulating thyroid hormones are known to exert specific effects on endochondral bone by promoting chondrocyte hypertrophy in the growth center [18], [19], [20]. The effects of thyroid hormone on membrane derived bone (e.g., bone comprising the cranial vault) have not been well studied.

There have been numerous reports of secondary craniosynostosis associated with hyperthyroidism [1], [2], [21], [22], [23], including the observation that maternal thyrotoxicoses is associated with development of sagittal and coronal craniosynostosis in the fetus or infant. In these cases skeletal maturation is advanced, measured by mineralization and bony thickness, suggesting that membrane bone may be more responsive to thyroid stimulation than endochondral bone. In contrast, early onset hypothyroidism results in widely patent sutures and fontanels suggesting a role for the homeostasis of thyroid hormone in the maintenance of patent sutures [5]. However, the biochemical effects of thyroid hormone on premature cranial suture fusion have yet to be elucidated, with the exception of identification of Insulin Like Growth Factors (IGF) presence at the calvarial suture where thyroid hormone influences the expression positively [24], [25]. To begin to address this deficit in our understanding, we performed a series of experiments to determine the effects of exogenous thyroid hormone exposure on cells derived from murine calvaria. The MC3T3-E1 cell line has been used previously to establish pathway changes relevant to the calvaria. As craniosynostosis is primarily a bone problem, bone is found where a patent joint should be, we focused our attention to osteogenic factors, as well as biochemical markers that have been previously associated with craniosynostosis. We hypothesized exogenous exposure to thyroid hormone would increase markers of osteogenesis in these mouse calvarial-derived cells.

## Materials and Methods

### Cells

The MC3T3-E1 murine osteoprogenitor cells, harvested from murine calvaria and purchased as a stock immortalized cell line (American Type Culture Collection (ATCC), Manassas, VA), were utilized in these studies on thyroxine (T4) (Sigma Aldrich, Saint Louis, MO) exposure. Cells were reconstituted and grown in a T-75 flask until transfer into T-175 flask. Cells were cultured in alpha minimum Eagles medium (αMEM) supplemented with penicillin/streptomycin (pen/strep) and 1% fetal bovine serum (FBS).

### Culture

For end-point assays, proliferation (MTS) and quantitative alkaline phosphatase (qALP), experiments were seeded in 96 well plates at a density of 4,000 cells per well. Treatments below were run in triplicate wells (averaged), and the studies were run in excess of triplicates (>3 plates per experiment, per time point). To test the effects of thyroxine hormone on mouse calvarial cells treatments were designed as follows 1) proliferation medium: control cells fed proliferation medium; and 2) Thyroxine reconstituted at concentrations of 1×10^−4^, 1×10^−5^, 1×10^−6^, 1×10^−7^, 1×10^−8^, 1×10^−9^, 1×10^−10^ mM. Cultures were treated for 3 or 7 days feeding every other day. Doses were chosen to mimic slight (subclinical) elevations in thyroid levels based on previous studies [26], [27], [28], [29].

For RNA extractions, experiments were set up in 6 well plates with 3 wells as control untreated cells, and 3 well thyroxine treated cells at the dose determined effective by the above endpoint assays. Seeding density was 50,000 cells per well. Samples in excess of triplicates (plates = samples) were utilized for studies at each time point. To test the effects of thyroxine hormone on biochemical markers related to osteoegensis of mouse calvarial cells treatments were set up as follows 1) proliferation medium: control cells fed proliferation medium or 2) Thyroxine 1×10^−6^ mM. Cultures were treated for 3 or 7 days feeding every other day.

### RNA Isolation

Cells were detached, lysates pelleted and RNA isolated using the Qiagen, RNeasy Plus Kit (Valencia, CA) following standard protocol. Quantity and quality of RNA was assessed using a Spectrophotometer (Nanodrop 1000, Wilmington DE). All ratios of absorbance at 260 and 280 nm were greater than 2.0.

### Osteoprogenitor Cell Proliferation Measures

Cell proliferation was determined by *Cell-titer 96 aqueous solution cell proliferation assay kit* (Promega, Madison, WI). After 3 or 7 days of treatment, cells were incubated with 20 ul of the solution added to each well. The absorbance was read at 490 nm and recorded (VersaMax, Molecular Devices, Sunnyvale, CA). Percent change in proliferation relative to baseline control measure (proliferation media treatment only) was assessed for each treatment.

### Quantitative Alkaline Phosphatase Measures

Cell differentiation was estimated using an Alkaline Phosphatase (ALP) activity assay. ALP is an early biochemical marker for osteoblast differentiation. After 3 or 7 days of treatment, medium was removed from cells, and cell lysis was performed using Triton ×100 at 0.01% (Sigma Aldrich, Saint Louis, MO). After 30 min of incubation at 4°C, deionized water and a p-Nitrophenyl phosphate solution was added to the lysis buffer. Three control wells containing no cells were also treated and served as blank controls to mathematically subtract the effects of the lysis buffer and water on final optical densities. Plates were incubated at room temperature in the dark for 30 min. The absorbance at 405 nm was recorded with a 96-well microplate reader. ALP activity was then calculated using the following formula: ((optical density – the mean optical density of the control wells)*total volume*dilution)/(18.45*sample volume). Percent change in differentiation relative to baseline control measure was assessed for each treatment.

### Quantitative Polymerase Chain Reaction for Markers of Osteoblastogenesis

Quantitative Polymerase Chain Reactions were run using a one-step kit for cDNA synthesis and gene expression (Life Technologies, Grand Island, NY). A master mix was made from nuclease free water, Taqman Master Mix, RT/Rnase inhibitor and commercially prepared probe/primer sets (**Table** **1**). 3 μl of RNA was added to the master mix for q-PCR for each gene product for each condition time (3, or 7 day) by treatment (media or levothyroxine). Expression data was normalized to 18 s expression by ΔΔCT. Quantitative data was compared by a time (3 or 7 day) by treatment (media or levothyroxine) 2×2 analysis. Genes of interest were *Runx2*, an early marker of osteogenesis, *Alp*, osteocalcin (*Ocn*), an osteoblast specific marker, and *Twist1*, shown to be important for stemness of cells and maintenance of the undifferentiated calvarial suture [30], [31].

**Table 1:** Quantitative Polymerase Chain Reaction Primer Data.

Gene Symbol	Gene Name	Primer Sequence	Reverse Primer
RUNX2	Runt Related Transcription Factor 2	Mm00501584_m1	GAGCCAGGCAGGTGCTTCAGAACTG
ALP	Alkaline Phosphatase	Mm00475834_m1	TGTGGCCCTCTCCAAGACATATAAC
OCN	Osteocalcin	Mm01741771_g1	CAGACAAGTCCCACACAGCAGCTTG
TWIST1	Twist Homolog 1	Mm00442036_m1	CAGGCCGGAGACCTAGATGTCATTG
HTRA1	HtrA serine peptidase 1	Mm00479892_m1	GGCAGAAGCCGGAGGGCTCAAGGAA
IGF1	Insulin Like Growth Factor 1	Mm00439560_m1	GCTTTTACTTCAACAAGCCCACAGG
PHEX	Phosphate regulating gene with homologies to endopeptidases on the X chromosome	Mm01166563_m1	CCACAATTTAGGGTCAATGGTGCCA
Col2a1	Collagen, Type II, alpha 1	Mm01309565_m1	GGTGACGACGGTGAAGCTGGGAAGC
TNF	Tumor Necrosis Factor	Mm00443260_g1	CCACGTCGTAGCAAACCACCAAGTG
FGF1	Fibroblast Growth Factor 1	Mm00438906_m1	AGCACATTCAGCTGCAGCTCAGTGC
FGF2	Fibroblast Growth Factor 2	Mm00433287_m1	AGAGCGACCCACACGTCAAACTACA
POR	P450 (cytochrome) oxidoreductase	Mm00435876_m1	GCAAGATCCAGACAACGGCCCCACC
DCN	Decorin	Mm00514535_m1	GATCCCTCAAGGTCTGCCTACTTCT
MMP9	Matrix Metallopeptidase 9	Mm00442991_m1	TCCAGTACCAAGACAAAGCCTATTT
DMP1	Dentin Matrix Protein 1	Mm01208363_m1	CTCTGAAGAGAGGACGGGTGATTTG
18S	18S ribosomal RNA	Mm03928990_g1	TACTTGGATAACTGTGGTAATTCTA

### Gene-Chip Array for Gene Expression

GeneChip® Mouse Gene 1.0 ST Array (Affymetrix, Santa Clara, CA) was utilized to determine target gene products that were dysregulated after 3 and 7 days of thyroxine exposure in our cells. This technology enables whole-genome gene-level expression studies for well-characterized genes. The GeneChip-brand array is comprised of over 750,000 unique 25-mer oligonucleotide features constituting over 28,000 gene-level probe sets. Initially, 150 ng of total RNA for control and thyroxine treated cell (1×10^−6^ mM) were examined for quality and quantity by bioanalyzer analysis (Agilent 2100, Santa Clara, CA). All sample rRNA ratios were greater than 2.5, and RNA integrity values were greater than 8. 250 ng of RNA was then used to prepare probes per sample. Samples were then compared by treatment (control or thyroxine) for 3 and 7 day time points. Fold changes greater than 2, or less than −2 are reported.

### Targeted Array for Gene Expression of Genes related to Osteogenesis

The Mouse Osteogenesis RT^2^ Profiler™ PCR Array (Qiagen, Valencia, CA) was utilized to further determine target gene products that were dysregulated after 7 days of thyroxine exposure in our cells. This array contains 84 genes that function in the development of the skeletal system as well as bone mineral metabolism. Purified RNA, 1 µG, was subjected to DNA elimination and then reversed transcribed to cDNA using the RT2 First Strand Kit (Qiagen,Valencia CA). The cDNA was then mixed with the RT2 SYBR Green Mastermix, and aliquoted into the wells of the array place. PCR was then performed (ABI 7300, Life Technologies, Grand Island New York) using standard protocol. Expression data between the control and thyroxine treated samples were compared by ΔΔCT. Fold changes greater than 2, or less than −2 were considered significant and reported.

### Quantitative Polymerase Chain Reaction Confirmation of Array Targets

Dysregulated targets of interest resulting from the Gene-Chip array or the targeted osteogenic array were chosen for confirmation by qPCR (**Table** **1**). Targets were chosen if they were a major component of the bone development pathway or have been implicated in dysregulation seen in craniosynostosis. Quantitative PCR was performed as described above using a one-step kit. Expression data was normalized to 18s expression by ΔΔCT. Quantitative data was compared by a time (3 or 7 day) by treatment (media or levothyroxine) analysis.

### Statistical Analysis

For proliferation and quantitative alkaline phosphatase, ANOVA was conducted for time in culture (3 or 7 day) by hormone treatment (thyroxine hormone doses between 10?-4 mol through 10?-10 mol). Intergroup differences were assessed using a post-hoc Bonferroni multiple comparison. Mean differences were considered significant if p<0.05. For arrays and qrt-PCR analysis mean fold changes were compared with a 2 fold change (upregulated or down regulated) being considered significant.

## Results

### Proliferation of Osteoprogenitor Cells is Affected by Thyroxine Treatment

**Figure 1:**
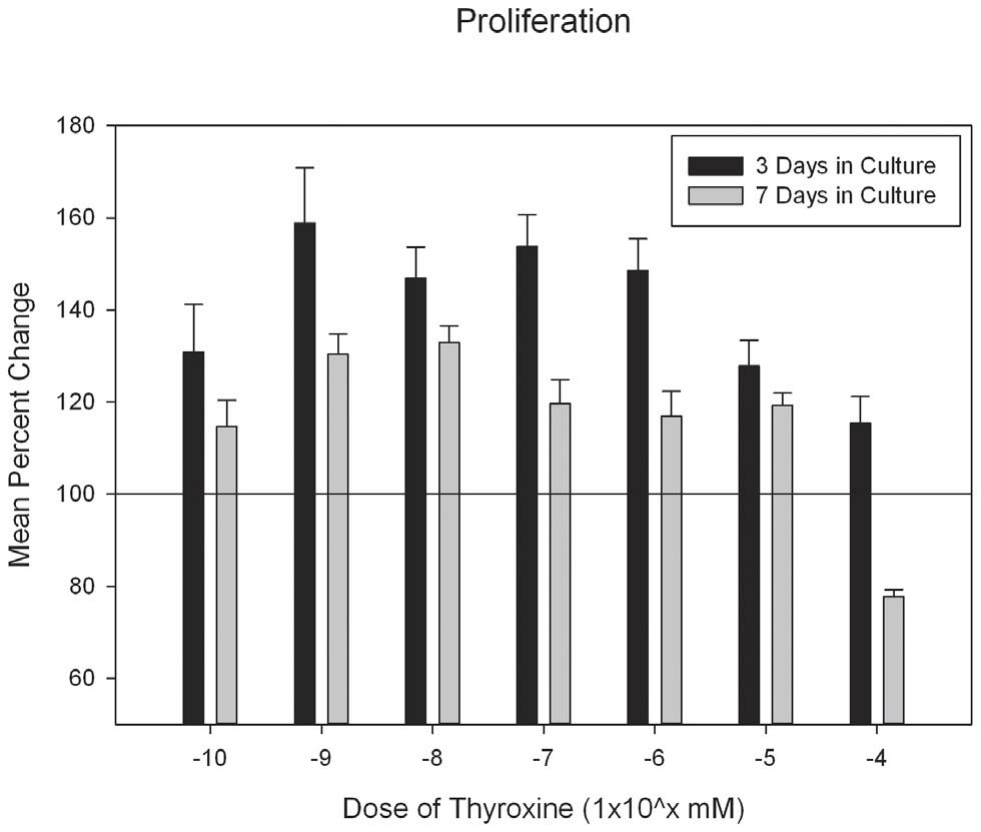
*Proliferation*: Cell proliferation assay after thyroxine treatment. Reference line indicates control (untreated) response and experimental group plotted as percent response compared to reference (error bars = standard error of the mean). Note the increases in proliferation after 3 and 7 days of treatment with the exception of the highest dose after 7 days.

MTS assay was conducted to investigate whether thyroxine treatment elicited changes in cell proliferation. An increase was observed in proliferation for the 3 and 7 day thyroxine treated assays, t = 9.818, p<0.001. To assess changes in cell proliferation after thyroxine treatment, culture data is presented as a percent change compared to baseline (proliferation media treated only). For the two-way comparison of days in culture by dose, there was no significant difference, p = ns. For days in culture there was a significant difference, F = 49.446, p<0.001 with the 3 day data showing a greater percent increase x = 140.30+/−3.74, compared to the cells treated for 7 days in culture, x = 115.94+/−3.25 (mean+/− standard error). For dose of thyroxine there was a significant difference, F = 12.331, p<0.001. Post hoc Bonferroni analysis revealed the 1×10^−4^ mol dose elicited a decrease in cell proliferation compared to baseline, p<0.05 and compared to the other doses which elicited increased proliferation (**Figure** **1**).

### Alkaline Phosphatase is Increased after Treatment with Thyroxine

**Figure 2:**
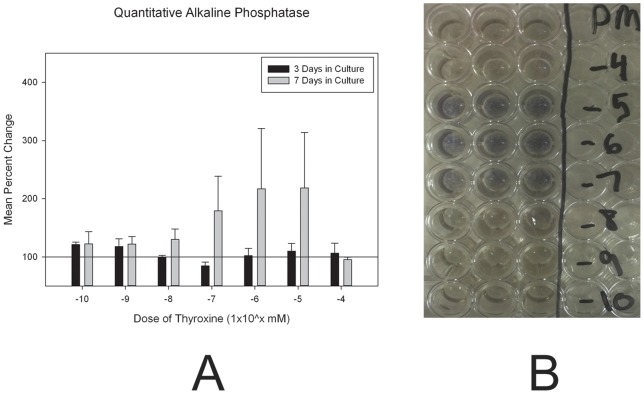
(a) *Quantitative Alkaline Phosphatase*: Alkaline Phosphatase activity assay indicative of cell differentiation. Reference line indicates control (untreated) response and experimental group plotted as percent response compared to reference (error bars = standard error of the mean). Note the great increases in response after 7 days of treatment for doses 10^−5^ through 10^−7^mM. (b) *Alkaline Phosphatase Stain*. Representative assay for Alkaline Phosphatase after 7 days in culture with thyroxine treatment. PM indicated proliferation media only. −4,−5,−6,−7,−8,−9,10 indicated thyroxine dose doses 10^−4^ through 10^−10^mM.

To analyze early osteoblast differentiation, quantitative alkaline phosphatase (ALP) analysis was performed. Thyroxine treatment elicited a greater response for ALP compared to baseline for 3 and 7 day treated assays, t = 2.669, p = 0.009. To assess changes in ALP after thyroxine treatment, culture data is presented as a percent change compared to baseline (proliferation media treated only). For the two-way comparison of days in culture by dose, there was no significant difference, p = ns. There were no significant differences in ALP by thyroxine dose, p = ns. There was a significant difference for ALP by days in culture, F = 4.657, p = 0.035, with a greater percent increase in ALP for cells treated with thyroxine for 7 days in culture, x = 154.93+/−21.75 compared to cells treated for 3 days, x = 105.88+/−4.31 (mean+/− standard error) (**Figure** **2a,b**).

### Quantitative Polymerase Chain Reaction Confirms Increased Expression of Osteoblast Markers

**Figure 3:**
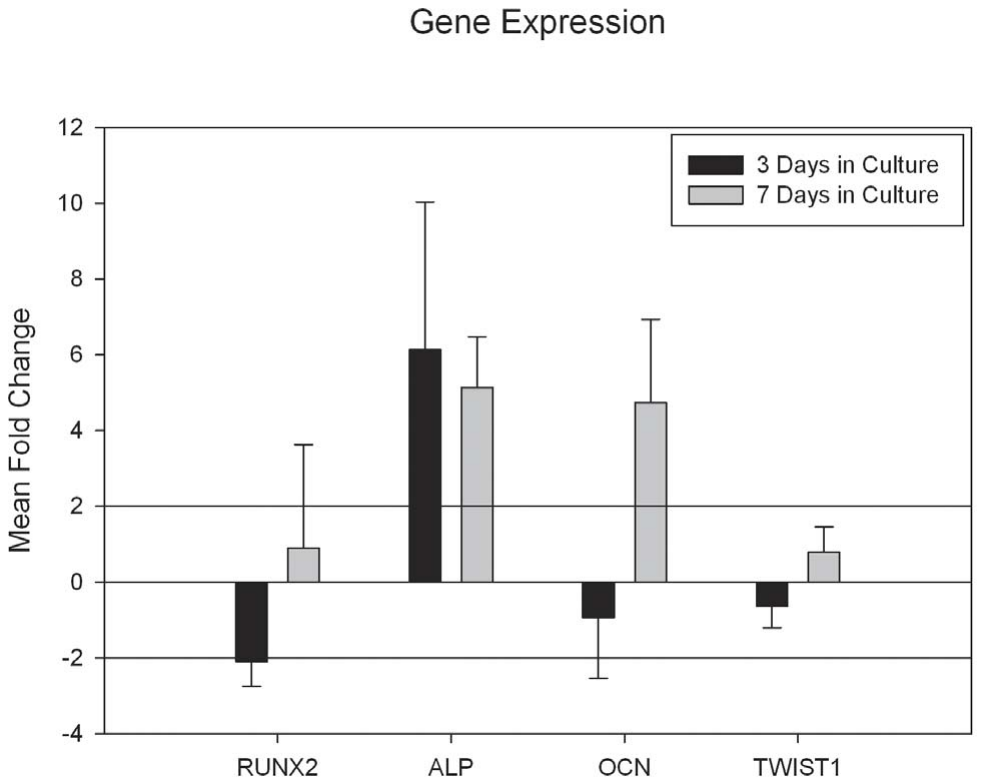
*Gene Expression*: Fold change mRNA for markers of osteoblastogenesis after thyroxine treatment for 3 or 7 days (error bars = standard error of the mean fold change). Note greater than two-fold up-regulations (reference lines) for alkaline phosphatase and osteocalcin.

Quantitative polymerase chain reaction was conducted to determine if mRNA expression occurred after thyroxine treatment. qPCR results were calculated using a ΔΔCT methodology. Results showed that after thyroxine treatment a greater than 2 mean fold changes resulted for expression in *Alp* after 3 and 7 days of in culture and *Ocn* exhibiting an increase at 7 days in culture. *Runx2* and *Twist1* appeared relatively stable exhibiting no significant mean fold changes (**Figure** **3**).

### Gene-Chip Array Confirms the Specific Effect of Thyroxine Treatment and Other Markers of Osteoblastogenesis

Gene-Chip Affymetrix Array was conducted to determine which genes would have expression changes after thyroxine treatment (GEO accession GSE47517). The results of the Gene-Chip array demonstrated 14 genes to be significantly up-regulated (greater than 2 fold expression change) and 4 genes to be significantly down-regulated (greater than −2 fold expression change) after 3 days of thyroxine exposure (**Table** **2**). For the up-regulated genesm those of great interest to bone development or metabolism were *Htra1* which regulates the bioavailability of Insulin like growth factors (*Igf*s). The array results further demonstrated 37 genes to be significantly up-regulated (greater than 2 fold expression change) and 11 genes to be significantly down-regulated (less than −2 fold expression change) after 7 days of thyroxine exposure (**Table** **3**). For the up-regulated genes, those of particular interest to bone development or metabolism were several isoforms of osteocalcin (also denoted *Bglap*), Insulin like growth factor (*Igf1*) and *Por* which is involved in P450 metabolism. For the down-regulated genes, Decorin (*Dcn*) was of interest to bone. *Dcn* acts to encode its protein which is a component of connective tissue, binds to type I collagen fibrils, and plays a role in matrix assembly.

**Table 2:** Gene-Chip MicroArray, Genes Dysregulated after 3 days of Thyroxine Treatment.

*Gene Symbol*	*Gene Name*	*Gene Symbol*	*Gene Name*
*Upregulated*		*Downregulated*	
Pcolce2	procollagen C-endopeptidase enhancer 2	Pappa2	pappalysin 2
Klf9	Kruppel-like factor 9	Lrrc17	leucine rich repeat containing 17
Ret	ret proto-oncogene	Zcchc5	zinc finger, CCHC domain containing 5
Slc27a1	solute carrier family 27 (fatty acid transporter), member 1	Igsf10	immunoglobulin superfamily, member 10
Htra1	HtrA serine peptidase 1		
Smpdl3b	sphingomyelin phosphodiesterase, acid-like 3B		
Spon2	spondin 2, extracellular matrix protein		
Tmod1	tropomodulin 1		
Igfbp4	insulin-like growth factor binding protein 4		
Zscan4a	zinc finger and SCAN domain containing 4		
Slc40a1	solute carrier family 40 (iron-regulated transporter), member 1		
Mir181b-1	microRNA 181b-1		
Gtf3c2	general transcription factor IIIC, polypeptide 2		
Fam43a	family with sequence similarity 43, member A		

**Table 3:** Gene-Chip MicroArray, Genes Dysregulated after 7 days of Thyroxine Treatment.

*Gene Symbol*	*Gene Name*	*Gene Symbol*	*Gene Name*
*Upregulated*		*Downregulated*	
Bglap2	bone gamma-carboxyglutamate (gla) protein	Lum	lumican
Tmod1	tropomodulin 1	Zcchc5	zinc finger, CCHC domain containing 5
Smpdl3b	sphingomyelin phosphodiesterase, acid-like 3B	Lrrc17	leucine rich repeat containing 17
Pfkfb3	6-phosphofructo-2-kinase/fructose-2,6-biphosphatase 3	ND6	mitochondrially encoded NADH dehydrogenase 6
Pcolce2	procollagen C-endopeptidase enhancer 2	AY036118	cDNA
Klf9	Kruppel-like factor 9	Enpp2	ectonucleotide pyrophosphatase/phosphodiesterase 2
Bdkrb1	bradykinin receptor B1	Snora73b	small nucleolar RNA, H/ACA box 73B
Tgtp1	T cell specific GTPase 1	Efhd1	EF-hand domain family, member D1
Htra1	HtrA serine peptidase 1	Fut11	fucosyltransferase 11 (alpha (1,3) fucosyltransferase)
9930013L23Rik	RIKEN cDNA 9930013L23 gene	Dcn	Decorin
Fam180a	family with sequence similarity 180, member A	Robo2	roundabout, axon guidance receptor, homolog 2 (Drosophila)
Spon2	spondin 2, extracellular matrix protein		
Ccl5	chemokine (C-C motif) ligand 5		
Ret	ret proto-oncogene		
Igf1	insulin-like growth factor 1 (somatomedin C)		
Npr3	natriuretic peptide receptor C/guanylate cyclase C		
Mir181b-1	microRNA 181b-1		
Bglap-rs1	bone gamma-carboxyglutamate (gla) protein		
Por	P450 (cytochrome) oxidoreductase		
Slc40a1	solute carrier family 40 (iron-regulated transporter), member 1		
Slc43a2	solute carrier family 43, member 2		
Trp53inp2	tumor protein p53 inducible nuclear protein 2		
A930018M24Rik	RIKEN cDNA A930018M24 gene		
Heg1	HEG homolog 1 (zebrafish)		
Fam43a	family with sequence similarity 43, member A		
9930111J21Rik2	RIKEN cDNA 9930111J21 gene		
Heg1	HEG homolog 1 (zebrafish)		
Ampd3	adenosine monophosphate deaminase 3		
Fam20c	family with sequence similarity 20, member C		
Sned1	sushi, nidogen and EGF-like domains 1		
Trdn	triadin		
Olfr767	olfactory receptor 767		
9930111J21Rik2	RIKEN cDNA 9930111J21Rik2		
Olfm2	olfactomedin 2		
Slc27a1	solute carrier family 27 (fatty acid transporter), member 1		
Adrb2	adrenergic, beta-2-, receptor, surface		
Itgbl1	integrin, beta-like 1 (with EGF-like repeat domains)		

### Targeted Osteogenic Array Confirms Positive Effect of Thyroxine on Osteoblastogenesis

A targeted osteogenic array was also conducted to further determine if genes associated with osteogenesis had expression changes after treatment with thyroxine. The results of the targeted osteogenic array after 7 days of thyroxine exposure demonstrated 22 gene products as up-regulated and 1 gene product was identified as down-regulated (**Table** **4**). The remaining gene products were found to have fold change of <2 and >−2, with no significant mean fold changes.

**Table 4:** Targeted Osteogenic MicroArray, Genes Dysregulated after Thyroxine Treatment.

*Gene Symbol*	*Gene name*
*Upregulated*	
Alpl	Alkaline phosphatase, liver/bone/kidney
Bmpr1b	Bone morphogenetic protein receptor, type 1B
Col10a1	Collagen, type X, alpha 1
Col11a1	Collagen, type XI, alpha 1
Col14a1	Collagen, type XIV, alpha 1
Col7a1	Collagen, type VII, alpha 1
Csf2	Colony stimulating factor 2 (granulocyte-macrophage)
Csf3	Colony stimulating factor 3 (granulocyte)
Dmp1	Dentin matrix protein 1
Fgf1	Fibroblast growth factor 1
Fgf2	Fibroblast growth factor 2
Fgf3	Fibroblast growth factor 3
Gdf10	Growth differentiation factor 10
Icam1	Intercellular adhesion molecule 1
Igf1	Insulin-like growth factor 1
Itga2	Integrin alpha 2
Mmp8	Matrix metallopeptidase 8
Mmp9	Matrix metallopeptidase 9
Phex	Phosphate regulating gene with homologies to endopeptidases on the X chromosome (hypophosphatemia, vitamin D resistant rickets)
Tnf	Tumor necrosis factor
Vdr	Vitamin D receptor
Vegfa	Vascular endothelial growth factor A
*Downregulated*	
Col2a1	Collagen, type II, alpha 1

### Confirmation of Dysregulated Array Targets

**Figure 4:**
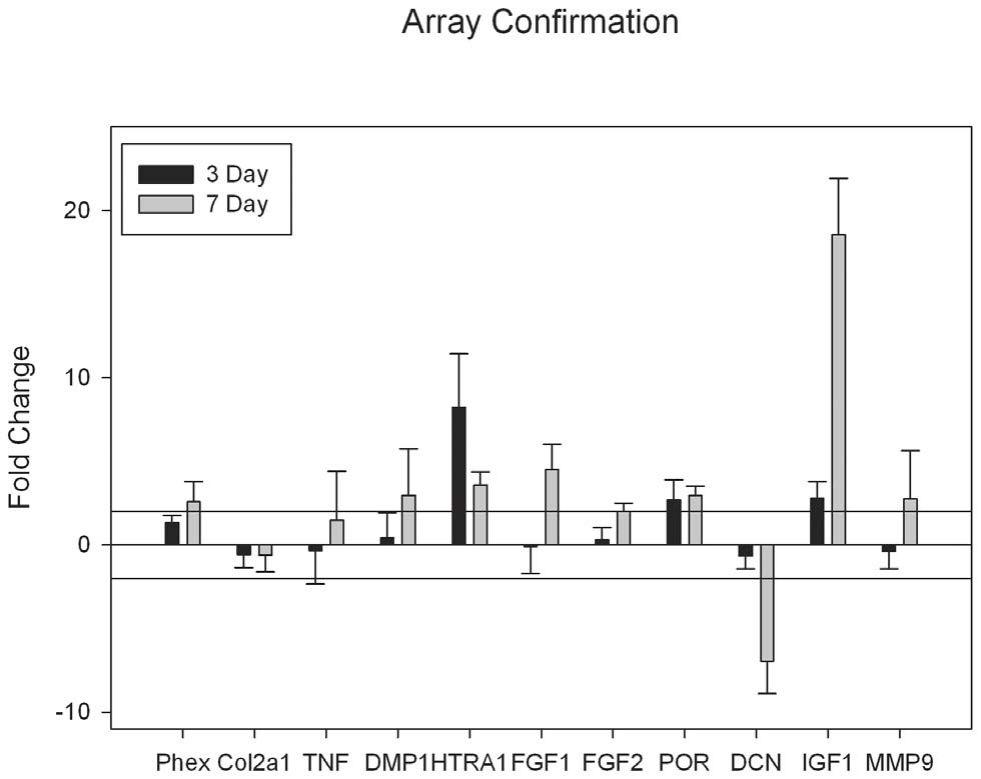
*Array Confirmation*: Confirmation of targets from gene arrays of interest. Expressed as fold changes mRNA after thyroxine treatment for 3 or 7 days (error bars = standard error of the mean fold change). Note two-fold changes (reference lines) or greater for targets: Phex, DMP1, HTRA1,FGF1,FGF2, POR, DCN, IGF1, and MMP9.

Of the targets identified by microarray, 11 gene products were chosen for qPCR confirmation. The gene products chosen were of particular interest to craniofacial bone development or craniosynostosis and were: *Phex*, *Col2a1*, *Tnf*, *Dmp1*, *Htra1*, *Fgf1*, *Fgf2*, *Por*, *Dcn*, *Igf1* and *Mmp9*. Results suggest significant dysregulations for 9 of these gene products after 3 or 7 days of thyroxine exposure (**Figure 4**).

## Discussion

It appears thyroid hormone may exert its effects on calvarial cells through increasing osteogenesis. Our data suggests an increase in proliferation at our early time point, and a bell-like curve response with our lowest and highest doses of thyroxine showing little effect typical of hormonal effects on cells. For alkaline phosphatase activity we observe an increase after 7 days in culture. These data are consistent with early increase in proliferation and later increase in differentiation suggesting that osteogenesis may be increased in these cells after exogenous thyroxine exposure. This relationship was confirmed with quantitative PCR which showed an increase in *Alp* expression at 3 and 7 days in culture and an increase in *Ocn* expression after 7 days in culture exposed to exogenous thyroxine. *Runx2* was not observed to be up-regulated. We would expect to see *Runx2* upregulation in cells destined to become osteoblasts as it is one of the earliest and an essential markers for osteoblasts. It may be that an earlier time point (perhaps 24 or 48 hours) would be necessary to capture its up-regulation.

Our genome wide array analysis identified 18 genes with significant changes in regulation after 3 days of exposure to thyroxine and 48 genes after 7 days of exposure to thyroxine. Although many genes were of interest, we selected genes to confirm by quantitative PCR in a greater sample (n = 3 for 3 days, and n = 3 for 7 days in culture) due to their relevance to bone and craniofacial biology. Among these genes were *Htra1* which was significantly up-regulated after 3 and 7 days in culture with thyroxine. This could have been predicted as HTRA1 is thought to regulate the availability of Insulin Like Growth Factor (IGF) [32]. Thyroid hormone has a positive effect on bone and bone cell growth through the IGF pathway [33], [34]. In addition, *Igf1* was significantly up-regulated after 7 days in culture exposed to thyroxine. In vivo, IGF1 is a mediator for the effects of growth hormone as well as a growth promoter for all cells in the body. An increase in the expression of *Igf* would translate to an increase in target cell proliferation and differentiation. Both of these genes were further confirmed by q-PCR to be significantly up-regulated at both 3 and 7 days in culture.

Two other genes from our genome wide array that showed significant dysregulation at 7 days were of interest. P450 reductase or *Por* is a membrane bound enzyme necessary for electron transfer in cell, from NADP to cytochrome P450 in microsomes [35]. Deficiency in POR has been linked to various diseases including disorders of steroidogenesis and Antley Bixler craniosynostosis syndrome [36]. However, we observed an upregulation in expression which may be suggestive of merely greater cell activity (proliferation/differentiation). Perhaps this is another example of a gene that required tight homeostasis to allow for normal growth and development of the calvaria. We further confirmed significant upregulation of *Por* at both 3 and 7 days in culture with thyroxine exposure.

Decorin codes for a small cellular proteoglycan which is part of connective tissue binding to type I collagen fibrils and playing a role in extracellular matrix assembly. Decorin is thought to regulate Transforming growth factor beta (TGFB) bioavailability and mutations in this gene have been linked to Marfan syndrome [37], [38]. Decorin has also been identified at the cranial suture with importance for suture formation and growth linked to its expression [39]. In addition, a study investigating the effects of FGF2 administration to Crouzon Syndrome (FGFr Autosomal Dominant Craniosynostosis Syndrome) patient derived parietal bone osteoblasts suggested an inverse relationship between FGF2 and DCN [40]. Overexpression of FGF2 has been implicated in craniosynostosis. We confirmed *Dcn* to be significantly down-regulated after 7 days in culture with thyroxine and as discussed below an upregulation of FGF2.

Our targeted osteoblast bone microarray after 7 days in culture identified 23 genes that were significantly dysregulated, 22 up-regulated and 1 down-regulated. *Igf1* was significantly up-regulated in our array and was confirmed as up-regulated. From these targets we chose to confirm 7 by qPCR, *Phex*, *Col2a1*, *Tnf*, *Dmp1*, *Fgf1*, *Fgf2*, and *Mmp9*. Dysregulation of *Col2a1* and *Tnf* was not confirmed. There are many reasons why a significant value could be obtained from a microarray analysis that is not confirmed by quantitative PCR, or vice versa including threshold reliability, fold change, intensity, and basic variability [41]. *Tnf* or Tumor necrosis factor was slightly up-regulated after 7 days in culture. This gene is important in apoptosis, mediates osteoclastogenesis and may have an inverse relationship with TWIST, with TWIST decreasing TNF mediated osteoclastogenesis [42], [43]. The slight upregulation seen here is most likely due to an increase in apoptosis of our cells. *Col2a1*showed a slight downregulation at both 3 and 7 days in culture. This gene allows for the production of type II collagen which provides structure and strength to connective tissues of bone, muscles, joints, and organs. Type II collagen is found primarily in cartilage [44], [45]. Chondrogenesis normally involves an increase in the expression of *Col2a1* and a decrease in *Col1a1*. Thus, this down-regulation of *Col2a1* may be an effect of promoting intramembranous ossification.

We did confirm increased expression in the genes *Phex*, *Dmp1*, *Fgf1*, *Fgf2* and *Mmp9*. PHEX is important in bone mineralization and deficiencies are linked to Vitamin D deficient rickets. It has been observed that *Phex* shows increased expression in osteoblasts especially during the transition from pre-osteoblasts to mature osteoblasts [46]. MMP9 or matrix metallopeptidase 9 is involved in the breakdown of extracellular matrix. MMP9 has been associated with differentiated osteoblasts in bone and the cranial suture. MMP9 is also a marker of osteoclast activity and remodeling [47]. DMP1 or Dentin matrix acid phosphoprotein is an ECM protein which is necessary for proper mineralization of bone. It regulates the expression of osteoblast specific genes and during osteoblast maturation the protein is phosphorylated and exported to the ECM where it orchestrates mineralized matrix formation [48]. In addition, in a mouse model that overexpressed DMP1, enhanced mineralization was shown at the cranial sutures [49]. Thus, the up-regulation of *Dmp1* at 7 days may be a target for future studies investigating molecular expression in suture fusion due to thyroid hormone exposure.

The fibroblast growth factors (FGFs) are a family of growth factors involved in angiogenesis, wound healing and development [31], [50], [51]. The fibroblast growth factor receptors have been implicated in syndromic and non-syndromic forms of craniosynostosis [52], [53], [54], [55]. We observed an upregulation in *Fgf1*,*2* and *3* after 7 days in culture in our targeted microarray. We were further able to confirm a significant upregulation for both *Fgf1* and *Fgf2* after 7 days in culture with thyroxine treatment. Both FGF1 and 2 have been shown to allow for growth of fetal and neonatal osteoblasts, and FGF2 is critical for calvarial development [50], [52], [53], [56], [57]. An upregulation in *Fgf2* can be indicative of osteoblast differentiation and had been linked to greater expression of downstream osteoblast targets, i.e. osteocalcin. FGF2 also aids in the control of the amount of apoptosis that occurs at the calvarial suture and the balance between undifferentiated and differentiated cells in the suture by promoting differentiation and apoptosis of more mature osteoblasts [50], [52], [56], [57], [58], [59]. FGFs may play a role in regulating IGF's effect on growth of calvarial cells, and in concert with TGFB1, FGF2 has been shown to be up-regulated in cranial sutures that are fusing [25], [45]. These up-regulated targets are also potential objectives for future studies investigating molecular expression in suture fusion due to thyroid hormone exposure.

Concerning translation to craniofacial biology our data speak to a specific relationship between calvarial cells and excess thyroid hormone exposure. We confirmed increases in markers of osteogenesis including *Alp*, *Ocn*, and *Mmp9*. We also confirmed targets that should be up-regulated in the presence of thyroid hormone, *Htra1* and *Igf1*, suggesting these cells are susceptible to thyroid hormone stimulation. In addition, the constituent decrease in *Dcn* and increase in *Fgf2* as well as *Fgf1* present possible targets in the pathway of effect for thyroxine exposure and osteogenesis of the calvarial cells, and perhaps suture. Overall, our targets suggest a possible interaction for those gene products associated with calvarial suture growth and homeostasis as well as craniosynostosis.
